# Weathering the storm: Effect of climate change on acute stroke care and stroke rehabilitation

**DOI:** 10.1002/pmrj.13218

**Published:** 2024-07-17

**Authors:** Erica M. Jones, Aardhra M. Venkatachalam, Nneka L. Ifejika

**Affiliations:** ^1^ Department of Neurology UT Southwestern Medical Center Dallas Texas USA; ^2^ Ross University School of Medicine Miramar Florida USA; ^3^ Department of Physical Medicine and Rehabilitation UT Southwestern Medical Center Dallas Texas USA

## Abstract

Climate change has deleterious effects on stroke recovery, disproportionately affecting populations with increased stroke incidence. These effects start prior to the acute care hospitalization, precipitated by environmental etiologies and are sustained throughout the life course of stroke survivors. Health care practitioners play a critical role in identifying these concerns and mitigating their impact through effective strategies at the patient level, interventions at the community level, and advocacy at the state and federal level. As the experts on improvement in function, quality of life, and the mitigation of disability, physiatrists have the opportunity to lead efforts in this space for stroke survivors and their caregivers.

## INTRODUCTION

**FIGURE 1:**
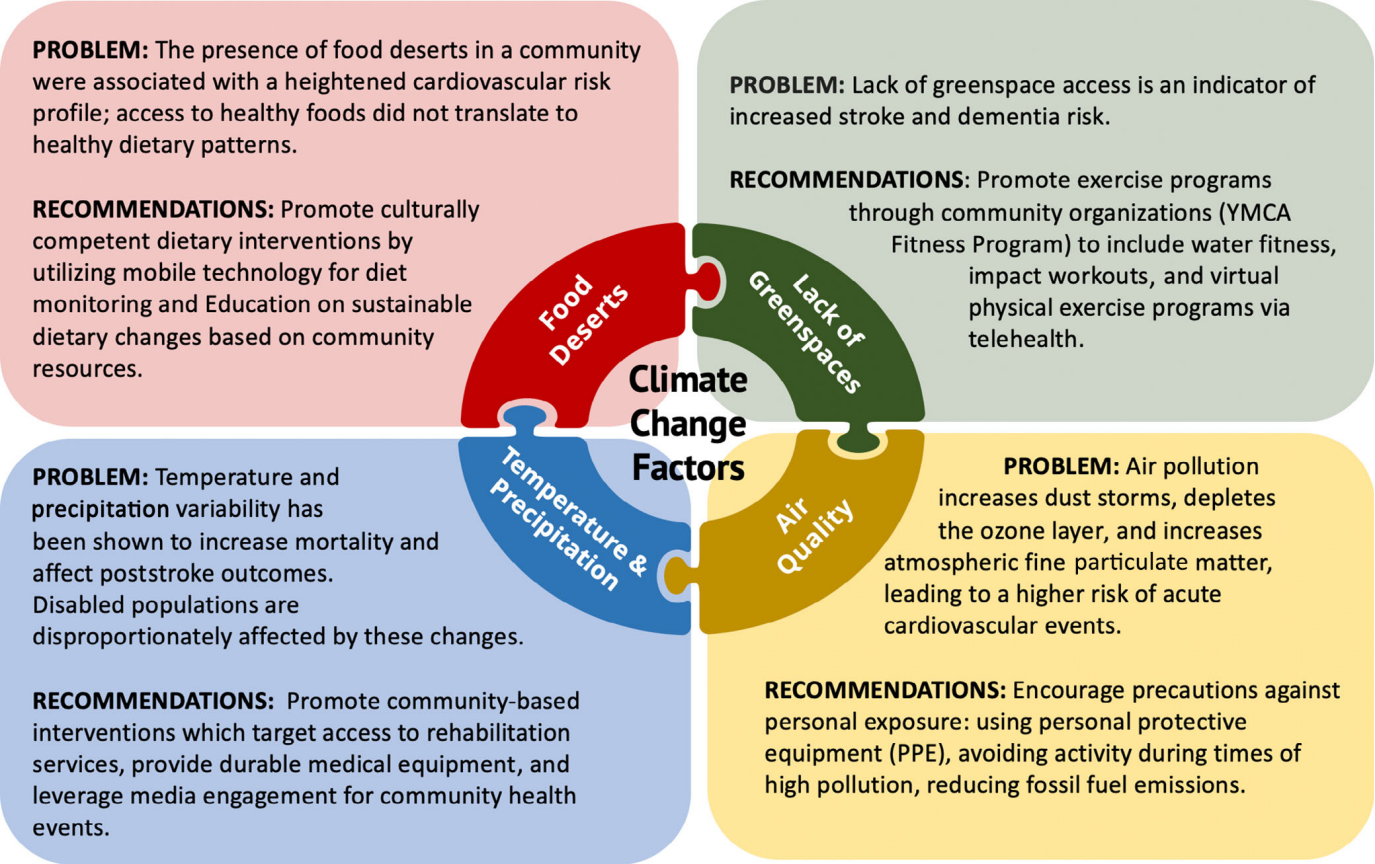
Opportunities to improve the effect of climate change on stroke survivors.

Substantiated by a *Lancet Planet Health* 2017 article, climate change disproportionately affects not only warmer, but poorer regions of the world.^1^ Centers for Disease Control and Prevention recommendations detail the importance of nutrition and dietetics and a good social environment, consisting of healthy food, access to physical activity, recreation, and smoke‐free settings, on the incidence of many conditions, including stroke.^2^ In this article we describe concerns and discuss strategies to reduce the impact of lack of greenspaces, food deserts, poor air quality, and variability in temperature and precipitation (Figure 1), particularly among stroke survivors with cognitive and mobility impairments.

## LACK OF GREENSPACES

Greenspaces, characterized as areas reserved for parks, gardens, and water features used for recreational or aesthetic purposes, have been shown to improve quality of life and offset the negative effects of urban development, including the urban heat island effect and surface water runoff. There is also a direct correlation between the lack of greenspaces and increased stroke incidence. In 2022, Cheruvalath et al. sought to determine whether greenspace was an independent risk factor for stroke, using the normalized difference vegetation index (NDVI) and a social determinant of health, area deprivation index (ADI), over a 3‐year period in a population cohort from Milwaukee, Wisconsin. NDVI and stroke risk were inversely correlated, with 19% lower odds of stroke for patients living in the highest greenspace quartile compared to the lowest quartile. Patients living in the most deprived ADI quartile had 28% higher stroke risk than those living in the least deprived ADI quartile. Non‐Hispanic Black patients lived in residential areas with lower greenspace, and higher state and higher national ADI compared to non‐Hispanic White patients.^3^

A 2022 population based prospective cohort study by Avellaneda‐Gomez et al. in Catalonia, Spain, analyzed the effect of air pollution and access to greenspace on ischemic stroke incidence. Individuals exposed to air pollution were at a greater risk of ischemic stroke while access to greenspace served as a *protective* factor to the same population in terms of reducing risk of ischemic stroke.^4^ Paul et al. in 2020 evaluated the effect of access to urban greenspace on dementia and stroke incidence using a retrospective, population‐based cohort for each study outcome. Using the Ontario Population Health and Environment Cohort in Canada, this study was the first to analyze this important relationship and determined both stroke and dementia risk was reduced with increased exposure to greenspace.^5^

In regions where local parks and recreation facilities are scarce, participating in home exercise programs provided by postacute care providers can prove difficult. Studies have shown that home‐based Health Enhancement Programs improved psychological health and adherence to rehabilitation recommendations and provided alternatives to patients who live in areas with limited greenspace.^6^ Many community organizations have structured activity programs, including the YMCA, which provides cardiovascular exercise, water fitness as well as low‐impact and chair‐level exercise programs. There are also virtual group exercise classes for stroke survivors who have mobility impairments that make travel difficult. The YMCA Enhance Fitness program has over 35,000 participants in the United States, of whom 35% report improvement in physical functioning and 53% reported reduced rates of depression.^7^

A significant concern for stroke survivors and their caregivers is related to safety. In areas with traffic congestion, the inability to walk quickly can heighten anxiety when endeavoring to transition from household to community ambulation. Future efforts to expand walkable greenspaces are detailed in the 2023 U.S. Environmental Protection Agency Green Streets, Green Jobs, Green Towns (G3) Initiative. The G3 Initiative supports the use of “green streets,” which incorporate vegetation, soil, and engineered systems (ie, permeable pavements) to clean and filter stormwater runoff from impervious surfaces, such as streets and sidewalks. Green streets improve the built and natural environment – an intermediate determinant of health inequities in the social determinants of health framework.^8^ Green streets help absorb carbon, improve air quality, provide green connections between parks and open spaces, improve pedestrian safety, and calm traffic.^9^

## FOOD DESERTS

Food deserts are geographic regions where residents have limited or no access to affordable, healthy food. Quantified by travel at least one mile in urban areas and 10 miles in rural areas, nearly 19 million people in the United States have limited access to a supermarket or grocery store.^10^ Inadequate consumption of healthy dietary options is strongly associated with chronic cardiometabolic illness, with food insecurity due to socioeconomic factors being the primary cause. The 2022 American Heart Association policy statement “Strengthening U.S. Food Policies and Programs to Promote Equity in Nutrition Security,” provided recommendations for food assistance policies to bridge the gap in nutrition security. The recommendations covered food accessibility, availability and affordability, food use, and dietary pattern stability. The recommendation sought to improve reach of food assistance programs by increasing enrollment and certification procedures, maintaining the dignity of participants by reducing the stigma of receiving nutritional assistance, and ensuring nutritional quality in distribution of resources.^11^

In 2017, Kelli et al. assessed the association between living in food deserts and an unfavorable cardiovascular disease (CVD) risk profile, characterized by measures including arterial stiffness, body mass index, high‐sensitivity C‐reactive protein, total cholesterol, low‐density and high‐density lipoprotein, and glucose, in 1421 participants residing in the Atlanta metropolitan area. Markers of oxidative stress, such as plasma levels of glutathione, cystine, and aniothiols, were also analyzed. Participant zip codes were cross‐referenced against the U.S. Food Access Research Atlas to determine food desert areas. The study concluded that although living in a food desert area was associated with greater unfavorable CVD risk and elevated markers of oxidative stress, the associations were more driven by participant income/socioeconomic status, a social determinant of health, and less driven by access to nutritional foods.^12^

The primary criteria for a food desert are related to proximity, however, access to healthy food does not always translate to changes in dietary patterns. In the Coronary Artery Risk Development in Young Adults (CARDIA) study, Boone‐Heinonen et al. found supermarket availability was unrelated to diet quality, vegetable and fruit consumption.^13^ This is of particular importance for stroke survivors as they transition from post‐acute rehabilitation to home. Although the U.S. Department of Agriculture Supplemental Nutritional Assistance Program, local community gardens, and regional civic organizations can help facilitate access to healthy food options at a lower cost, uptake and buy‐in are an essential part of improving the nutritional status of stroke survivors. Culturally competent dietary interventions are an innovative way to bridge this divide. Previous studies using a combination of mobile health technology for dietary monitoring, along with education on dietary changes and structured programs that respect and honor patients with stroke and their caregivers, have resulted in effective weight management, improved dietary consumption, and decreased poststroke depression.^14^, ^15^

## AIR QUALITY

Air pollution is a significant public health concern, affecting approximately 9 out of 10 people who reside in cities worldwide.^16^ It is a substantial contributor to global morbidity and mortality, causing up to an estimated 7 million premature deaths annually. Climate change is expected to exacerbate the health effects of air pollution by changing the dispersion of the most common pollutants and increasing the formation of ozone. Increasing wildfires and dust storms are also projected to contribute to this impact.^17^ A study by Wellenius et al. showed that daily changes in levels of ambient fine particulate matter (PM) air pollution (PM <2.5 μm in diameter [PM_2.5_]) have been associated with higher risk of acute cardiovascular events, excess hospitalizations, and deaths. The study conducted a retrospective chart review of patients in the Boston area confirmed with a diagnosis of ischemic stroke and measured exposure to PM_2.5_. There was a significant increase in risk for ischemic stroke associated with greater particulate matter exposure, with the greatest risk within 12 to 14 hours of exposure to PM_2.5_ when assessing traffic‐related pollution.^18^ In a meta‐analysis of 94 studies on short‐term air pollution exposure, there was a strong temporal relation to stroke mortality and hospital admission for stroke. It is notable that most of the studies included in this analysis were from high‐income countries, although low‐ and middle‐income countries are projected to have an increasing share of the global burden of stroke incidence and mortality.^19^ Low‐ and middle‐income countries are also projected to have increasing air pollution due to industrialization and the energy demands of the approximately 85% of the world's population that live in these developing nations.^20^

Without successful mitigation efforts to reduce the accumulation of air pollutants, more people will have chronic, long‐term exposure. Increases in long‐term air pollutant exposure have been associated with an increased risk of stroke mortality and increased risk of incident stroke.^21^, ^22^, ^23^ Even after admission for stroke, long‐term exposure to air pollution was associated with up to a 2.0% increased risk of 30‐day readmission.^24^ The pathophysiology underlying the risk conveyed by air pollution is not yet well understood but chronic inflammation from pollutant exposures has been associated with atherosclerosis, hypertension, endothelial dysfunction, and cardiac arrhythmias, which are suspected to mediate the risk of cerebrovascular disease.^25^

In 2005, Hashem at the Lawrence Berkeley National Lab attributed as much as 20% of population weighted smog concentrations to “urban heat islands”; cities that have been paved over do not receive the natural cooling effect of vegetation.^26^ Green infrastructure, in the forms of publicly or privately owned parks and green streets, can decrease the size of urban heat islands by reducing air temperature, the demand for air conditioning, and emissions from power plants to meet cooling needs. Furthermore, green spaces directly reduce air pollution through the filtration and absorption of PM.

The stroke risk reduction benefits of decreasing exposure to air pollutants have not been thoroughly investigated, but precautions are recommended to reduce personal exposure. These efforts can include avoiding outdoor activity during times of high pollution, using air filters or purifiers in the home, or wearing personal protective equipment in areas where exposure is likely. Public policies reducing fossil fuel emissions have been supported by various health organizations as a method to address this issue on a population level.^25^

## TEMPERATURE AND PRECIPITATION

Climate change and the effects on global weather patterns have direct and indirect impacts on stroke survivors. Increasingly frequent heatwaves, storms, and floods and the disruption of food systems have a magnified impact on stroke survivors with mobility issues, especially when dependent on community‐based transportation services, and those on fixed income, who may have difficulty affording housing in areas under less of a threat. Temperature and precipitation extremes influence social determinants of health, including built and natural environment, public health and health care, and structural determinants of health inequities.^8^

Chu et al. performed a cross‐sectional study from 896 hospitals with data from Get With The Guidelines‐Stroke, examining the effect of climate variables on stroke outcomes.^27^ Nine U.S. climate regions – defined by the National Climatic Data Center – were examined, with the primary outcome of in‐hospital mortality. Climate variables included 7‐day average minimum and maximum temperatures and 7‐day average precipitation, with the Northeastern United States as the reference group. In this study of over 450,000 patients with ischemic stroke, precipitation and temperature showed significant associations with in‐hospital mortality, a 5°C increase in minimum temperature or an increase in precipitation of 10 mm yielded an odds ratio of 0.95 for in‐hospital mortality. In addition, temperature increase yielded an odds ratio of 1.02 for the secondary outcome of home discharge post stroke. Overall, warmer and wetter weather conditions were associated with better outcomes.

The findings that warmer regions of the United States with more precipitation have lower poststroke mortality is encouraging, however, cities in the southern and southeastern United States have experienced increased intensity of severe weather, resulting in access to care limitations for vulnerable populations, including stroke survivors. Social inequities after Hurricane Katrina in 2007 resulted in a substantial expansion of the literature on environmental justice, and the impact of large‐scale flooding on the health and well‐being of New Orleans residents.^28^ In August 2017, Hurricane Harvey rainfall was estimated as a 3000 to 20,000‐year flood event, disproportionately affecting vulnerable populations in Houston, Texas, including the disabled.^29^ Disabled populations are disproportionately exposed to environmental health hazards, feeling abandoned by health care systems due to service marginalization.^30^ To address these concerns, community‐based interventions to facilitate access to care for stroke survivors have been implemented in flood prone regions. The Rehabilitation Services Volunteer Project in Houston is a nonprofit organization that provides clinical care, rehabilitation services, and durable medical equipment to persons with disabilities who are uninsured or underinsured (https://rsvptexas.org/services/). The Stomp Out Stroke initiative is an annual community‐based public health event, which leverages multimedia engagement to created patient‐centered cerebrovascular health interventions. In 2018 and 2019, internet‐based channels using the Stomp Out Stroke webpage, social media, *#stompoutstroke*, television, iQ radio, and web‐based news reached approximately 849,731 people in the Houston area.^31^ Over 2500 health screenings were conducted during two large‐scale events, averaging four health screenings per minute.

## CONCLUSIONS

What recommendations can physiatrists, therapists and other stroke rehabilitation and recovery providers give to patients who reside in regions disproportionately affected by climate change? First, disparities attributed to climate change overlap with disparities attributed to social determinants of health, detailed in the National Institute of Neurological Disorders and Stroke 2023 Health Equity Recommendations.^8^ Second, it is imperative that persons affected by stroke and their families have an understanding that recovery does not solely exist within the purview of health care providers, including physicians, nurses, and therapy services. Stroke recovery exists in society – in the home, the community, the faith organization, the work environment, and beyond. Effective communication with stroke survivors and their caregivers on these four topics provides the ability to address their concerns and to facilitate the provision of available resources. Educating patients, health care providers, and communities on climate change as a growing public health concern in patients with cerebrovascular disease is a key component of galvanizing national support. Finally, the programs in this review can be expanded. This is an open call for stroke rehabilitation and recovery providers with passion, interest, and bandwidth to take the helm and make a difference.

## FUNDING INFORMATION

Dr Ifejika's current work: UT Southwestern/Texas Health Resources Clinical Scholar Award (#4).

## DISCLOSURE

None.
